# Plasma membrane calcium-ATPase isoform four distribution changes during corneal epithelial wound healing

**Published:** 2010-11-02

**Authors:** Ernest F. Talarico

**Affiliations:** Department of Anatomy & Cell Biology, Indiana University School of Medicine-Northwest, Gary, IN

## Abstract

**Purpose:**

Plasma Membrane Calcium-ATPases (PMCAs) are integral membrane proteins essential to the control of intracellular Ca^2+^ concentration. In humans, four genes encode PMCA proteins termed PMCA1-PMCA4. PMCA4 is the major PMCA isoform expressed in human corneal epithelium (hCE); however, little is known about its role. The present study documented expression of PMCA4 in rabbit CE (rbCE) and followed the distribution of PMCA4 during CE wound healing in a rabbit (rb) model.

**Methods:**

Reverse transcriptase PCR using PMCA4 isoform gene-specific primers that flanked alternative splice site A was used to examine the presence of PMCA4 mRNA in rbCE. Protein expression was assessed by immunoblotting using panPMCA- and PMCA4-specific antibodies. Immunocytochemistry was employed to examine PMCA immunolocalization in frozen, formaldehyde-fixed sections of control and wounded rb corneas. In wound healing studies, circular, 6-mm diameter corneal wounds were produced in the central CE using the n-heptanol technique. The distribution of PMCA4 in CE was examined by immunohistochemical staining of frozen sections using PMCA4 isoform-specific antibody at 6-, 24-, 36-, and 48 h post-injury. siRNA_PMCA4_ was used to transfect telomerase-immortalized human corneal epithelial (hTCEpi) cells. Cell cultures were wounded 48 h after transfection, and the wound area was measured at 0 h and at 3 h intervals post-wounding.

**Results:**

Direct sequencing of PCR DNAs documented the presence of PMCA4 transcripts in rbCE and showed that the splice variant at site A was PMCA4x. Immunoblot analysis for PMCA4 detected an intense band at approximately 160 kDa and a faint band at approximately 142 kDa. Immunohistochemistry with the panPMCA antibody demonstrated strong immunoreactivity (IR) in all layers of uninjured rbCE. Immunohistochemistry with a PMCA4-specific antibody demonstrated a similar pattern of intense IR along the plasma membrane of cells in all layers of CE, except for the notable absence of PMCA4 IR along the basal cell membranes adjacent to the stroma. The pattern of PMCA4 IR changed following wound healing. During the lag phase of corneal epithelial wound healing, PMCA4 IR was seen mostly on apical plasma membranes of basal cells near the wound margin, with little staining of basal plasma membranes. During the migration phase (24 h), PMCA4 IR was found mostly on basal cell membranes adjacent to the stroma. At 6 h and 24 h following wounding, PMCA4 IR of the cytoplasm was increased compared to control eyes. After closure of the denuded area and stratification, PMCA4 IR was again primarily found along the apical and lateral plasma membranes of basal cells and was again absent from basal cell membranes adjacent to the stroma; PMCA4 IR of the cytoplasm was also similar to that observed in control eyes. siRNA_PMCA4_ transfected hTCEpi cells failed to seal the wound area, whereas wounds in control cultures transfected with a scrambled construct were completed healed.

**Conclusions:**

PMCA4 is strongly expressed in rabbit CE and its immunolocalization exhibits marked changes in distribution during the wound healing process. Knockdown of PMCA4 expression in hTCEpi cells decreases wound healing. Present findings suggest that PMCA4 redistribution could function as one factor in mediating calcium-regulated events necessary for cell migration in regenerating CE.

## Introduction

The corneal epithelium (CE) after artificial wounding provides a valuable model to study the migration of stratified epithelial cells in vivo. The CE is a nonkeratinized stratified squamous epithelium that covers the anterior surface of the cornea [1-4]. It consists of 2–3 layers each of superficial squamous cells and intermediate wing cells, and a single layer of basal cells next to the corneal stroma [5]. Basal cells are the only corneal epithelial cells capable of proliferating, and provide a continuous supply of new cells to replace terminally differentiated cells lost from the superficial layer during desquamation and eye blinking [5-7].

CE exhibits a highly developed ability to repair itself following wounding; thus, providing a mechanism to quickly re-establish integrity of its critical barrier functions [8]. The process of corneal epithelial wound closure is essentially a biphasic process comprised of an initial latent phase and a subsequent cell migration phase [9,10]. The latent phase lasts approximately 4–6 h, during which time cellular mitosis and DNA synthesis are nearly halted [11-13] and epithelial cells at the wound margin undergo extensive cellular and subcellular reorganization as they prepare to migrate into the defect. By 6 h post injury, basal cells near the wound margin have lost their columnar appearance and have broken many of their hemisdesmosomal attachments to the basal lamina [10]. In addition, cells at the wound margin become exceedingly thin and exhibit filopodia and ruffled boarders typical of migrating cells [10]. The latent phase is then followed by a migration phase that is characterized by a burst of proliferative activity in more peripheral basal cells [11,13], and concurrent migration of cells at the wound margin into the denuded area. The cells at the leading edge pull more peripheral cells behind them as a continuous epithelial sheet until the wound defect is covered by a monolayer of cells [14-17]. Increased proliferative activity continues above basal level for approximately 2–7 days until the full thickness of the CE is re-established [18].

The process of CE wound healing is complex and involves a coordinated sequence of physiologic events including cell migration, proliferation and differentiation [18,19], all of which depend on calcium-mediated processes. For example, both intracellular and extracellular Ca^2+^ concentrations regulate integrin-mediated adhesion during cell migration [20]. Ca^2+^ and the calcium-binding protein, calmodulin (CaM), are needed for cells to enter the synthesis (S) phase of proliferation [21-24]. Cell differentiation and adhesion are also dependent upon [Ca^2+^]_i_ [20,25].

One of the principle enzymes regulating intracellular Ca^2+^ concentration is plasma membrane Ca^2+^-ATPase (PMCA). PMCAs are present in the surface membranes of cells and extrude Ca^2+^ against very large concentration gradients [26,27]. In humans, four genes known as *ATP2B1-ATP2B4* encode PMCA proteins termed PMCA1-PMCA4, respectively [28-32]. PMCA1 and PMCA4 are expressed ubiquitously [30,33-36], whereas PMCA2 and PMCA3 are distributed in a tissue-specific fashion with PMCA2 expressed preferentially in brain and cardiac muscle and PMCA3 in brain and skeletal muscle [37,38]. PMCA isoform diversity is further increased via alternative splicing of primary RNA transcripts [30,33,38,39] that could result in more than 25 distinct proteins [38,40]. This diversity and the complex nature of temporal and spatial isoform expression suggest that the physiologic function of PMCAs extends beyond simply maintaining low [Ca^2+^]_i_ [28,36,37,39].

It has been shown that during the migration phase of mouse CE wound healing, PMCAs redistribute from plasma membrane caveolae to membranous structures in the cytoplasm. PMCAs then returned to the plasma membrane when the defect was healed [41,42]. This is significant in that many of the processes that occur during wound healing involve Ca^2+^ as messenger or trigger, including synthesis of proteins and glycoproteins [43], glycogen metabolism [44], and actin filament reorganization and podial extension [9,45,46]. Redistribution of PMCAs suggests that regional changes in [Ca^2+^]_i_ may occur during the wound healing process, and that changes in [Ca^2+^]_i_ could function in the regulatory programs that direct changes in cell motility, morphology and adhesion.

Prior studies in our laboratory have determined the expression of PMCA splice variants and their distribution in native human corneal epithelium [47,48]. These results showed that PMCA4 was the predominant isoform in human CE, and that PMCA4 was localized to the epithelial cell plasma membranes in all layers of CE.

Critically, there was lack of PMCA4 immunoreactivity along the portion of the plasma membranes of basal cells adjacent to the underlying stroma. Because basal cells in CE are the only cells in CE capable of mitosis and CE wound repair, and the localization of PMCA4 differs in basal cells compared to the other cell types (i.e., wing and squamous) that comprise CE, we hypothesized that PMCA4 would be a good “candidate PMCA” to study. Thus, in the present work, we investigated the localization of PMCA4 during CE wound healing in a rabbit (rb) model. We report here that during CE wound healing, PMCA4 redistributes in the plasma membrane of CE cells at the wound margin and returns to its normal distribution soon after the wound is closed. We hypothesize that PMCA4 redistribution is a factor in mediating calcium-regulated events necessary for cell migration in regenerating CE.

## Methods

Experiments were conducted according to the guidelines of Indiana University School of Medicine and in compliance with federal regulations governing the use and protection of animal subjects in research. A total of 14 male, New Zealand albino rabbits, approximately 4–5 pounds each, were purchased from Kuiper Rabbit Ranch (Gary, IN) for use in this study.

### Immunoblotting

CE from three normal rabbits was removed by mechanical scraping with a No. 10 scalpel blade while the cornea was immersed in ice-cold 0.1 M PBS (0.084 M Na_2_HPO_4_, 0.016 M KH_2_PO_4_, 0.9% NaCl). Sample preparation, electrophoresis, and immunoblotting were done as previously described [47]. Briefly, cells were placed into a conical centrifuge tube, suspended by gentle pipetting, and centrifuged for 7 min. The cell pellet was resuspended and washed in PBS. An aliquot was removed for determination of total protein concentration, and the remaining suspension was solubilized in electrophoresis sample buffer. CE samples and molecular weight standards were resolved by SDS-PAGE using a Laemmli system with an 8% gel. Proteins were transferred to PVDF membranes (Immobilon-P; Millipore, Burlington, MA) by semi-dry electroblotting.  Blots were wet with MeOH, washed using a high salt Tris Buffered Saline Solution (HTBS), and blocked at room temperature (RT).  After a wash in HTBS, blots were reacted with primary antibody RT.  The blots were then washed, blocked a second time, and incubated for RT with secondary antibody. The blots were then washed four times in HTBS, and incubated for 30 min at RT in CDP-Star chemiluminescent substrate (Applied Biosciences, Bedford, MA) in order to visualize the cross-reacting bands. The chemiluminescent signal was captured using a Kodak Image Station 440CF (Kodak, Rochester, NY).

### Sequence determination of PMCA4 alternative splice variants

CE removed by mechanical scraping was stored in RNA*Later*^TM^ (Ambion, Austin, TX). The tissue was weighed and total RNA was extracted using an RNAqueous-4PCR Kit (Ambion). The RNA concentration was determined by spectrophotometric (260 nm versus 280 nm) analysis.

Total RNA extracts were used for reverse transcription (RT) using the SuperScript^TM^ First Strand Synthesis for RT–PCR Kit (Invitrogen, Carlsbad, CA). PCR was done using the High Fidelity PCR Master Kit (Roche, Roche Diagnostics, Mannheim, Germany) with 1 μl cDNA template and 1 μl 400 nM each of forward and reverse PMCA4 isoform gene-specific primer. The primer set for PMCA4 (Invitrogen, Life Technologies, Grand Island, NY) flanks alternative splice site A [38,49], and has been successfully used in prior research in our laboratory [50,51]. The forward primer, pmca4apa has the sequence 5′-TAC TCT CTT GGG GGT CAA TGA-3′, and a starting position at residue 1,736. The reverse primer, pmca4ama, has the sequences 5′-CCA TGG TCT GCG ATT TAT CAC A-3′, and a starting position of 2,066. Reactions were amplified using a GeneAmp PCR System 2000 (Applied Biosciences). The following cycling conditions were employed: initial denaturation at 94 °C (2 min), 10 cycles of 94 °C (15 s), 55 °C (30 s) and 72 °C (40 s), 25 cycles of 94 °C (15 s), 55 °C (30 s) and 72 °C (40 s increased by 20 s per cycle), then a final extension at 72 °C (7 min), and a hold at 4 °C [51]. Aliquots of PCR products and PCR markers (Promega, Madison, WI) were electrophoresed in 2.0% agarose gel in 1X TAE Buffer using Rapid Acrylamide Gel Electrophoresis (RAGE) at 210 V for 8 min, and visualized using an Ultraviolet Transilluminator (Ultraviolet Products, Upland, CA) connected to a Kodak Electrophoresis Documentation/Analysis Imaging system (EDAS290; Kodak).

PCR DNAs were purified using the Wizard^®^ PCR Preps DNA Purification System (Promega) and Vac-Man^®^ Jr. Laboratory Vacuum Manifold (Promega). Purified samples of PMCA PCR DNAs were submitted for direct sequencing to the Cancer Research Center DNA Sequencing Facility at the University of Chicago. The sequences were analyzed using Chromas V2.11 (Technelysium Pty. Ltd., Tewantin QLD, Austrailia). Edited sequences were aligned and translated for determination of amino acid sequence using the Baylor College of Medicine Search Launcher-Multiple Sequence Alignment Program (Baylor College of Medicine, Baylor, TX). DNA and amino acids sequences were compared to known sequences using the National Center for Biotechnology Information Nucleotide BLAST and Protein/Translated Sequence Blast, respectively, and ExPASy was also used for the determination of translated sequences.

### Corneal epithelial wounding protocol

Rabbits were deeply anesthetized with 2.5% Pentothal (Fort Dodge® Animal Health, Fort Dodge, IA) administered via a lateral marginal ear vein. A unilateral circular wound was produced in the central CE of the experimental (left) eye using the n-heptanol technique [52]. Briefly, the eye was proptosed by applying gentle pressure behind the globe and a 6.5 mm diameter Wratten No.1 filter paper disc saturated with n-heptanol was gently pressed against the central cornea for 30 s. The disc was then removed and the cornea was rinsed with 10 ml of warm, sterile saline. This protocol produces a well defined, circular epithelial defect with little or no damage to the underlying corneal stroma [52]. Prior work has shown that corneal wounds produced by this method typically heal completely within 2–3 days [16,52]. The control [53] eye of each experimental animal was treated in an identical fashion except that 0.1 M PBS (0.084 M Na_2_HPO_4_, 0.016 M KH_2_PO_4_, 0.9% NaCl) was substituted for the n-heptanol. The animals were examined at regular postoperative intervals for any signs of squinting, ocular redness, or infection.

At 6, 24, 36, and 48 h post-wounding (there were 3 animals at each time point, respectively, the third animal for the 48 h post-wounding time point had to be prematurely sacrificed due to eye infection.), animals were euthanized with 1.5 ml of Sleepaway (Sodium Pentobarbital 26%, isopropyl alcohol 7.8%, Fort Dodge^®^ Animal Health). Wounded and control corneas were harvested from each animal, placed in ice-cold 0.1 M PBS, and cleaned of all excess tissue except for a thin rim (~1.0 mm) of sclera. The center of each cornea was marked and the corneas were cut into quadrants using a sterile No. 10 scalpel blade. The quadrants were immersion-fixed for 30 min at room temperature in 4% paraformaldehyde containing 0.2% picric acid and 2% sucrose, pH 7.3, and then passed through a series of sucrose solutions (5%, 10%, 20%, and 30% sucrose in 0.1 M PBS) for at least 1 h each at 4 °C. Tissues were stored in ice-cold 30% sucrose-0.02% sodium azide containing 0.1 M PBS until sectioned.

### siRNA experiments

hTCEpi cells were provided by Jerry W. Shay, Department of Cell Biology, UT Southwestern Medical Center (Dallas, TX). These cells have been characterized for use in CE cell culture experiments [54]. hTCEpi cells were seeded onto primaria-coated, 6-well plates at a concentration of 3.0 × 10^4^ cells/well and grown to form confluent monolayers in KBM-2 media (KBM-2 Bullet Kit, #CC3170; Lonza, Walkersville, MD). Media was aspirated and “naked” siRNA transfection was done in separate wells using a 30 nM final concentration in 2.0 ml of KBM-2 media of siRNA_PMCA4_ construct for Exon 17 of PMCA4 (Ambion) and a siRNA_Scrambled_ construct (Ambion) with no known homologous targets in human CE cells. The sequence (5′→3′) for the siRNA_PMCA4_ construct was 5′-GCA CUA UAC CAU UGU UUU Utt-3′ (sense) and 5′-AAA AAC AAU GGU AUA GUG Ctg-3′ (antisense). siRNA_Scrambled_ construct was supplied in the siRNA Transfection Kit II (Ambion). The final concentration of siRNA used was determined as described in the siRNA Transfection Kit. At 48 h post-transfection, cultures were wounded by dragging a sterile 200 μl pipette tip across the surface of the well, and the cultures were washed 3 times with KBM-2 media, and then 2.0 ml of KBM-2 media was added to each culture. Photomicrographs were taken at 0 h and at 3 h intervals thereafter for a total of 30 h. The wound area measured using AxioVision Software R6.2 (Carl Zeiss, Inc., Göttingen, Germany*).* The calculated wound area was normalized to 0 h, and plotted as wound area versus time post-wounding. A total of 9 wells each for siRNA_PMCA4_ and for siRNA_Scrambled_ transfected cultures were done. Statistical analysis for standard error and *t*-test with p-values were done using Excel 2010 (Microsoft Office, Microsoft Corporation, Redmond, WA).

### Immunohistochemistry

Immediately before sectioning, a 1 mm – wide rectangular piece of tissue was cut from one edge of each quadrant and placed into OTC Compound (Tissue-Tek^®^; Sakura Finetechnical Co., Tokyo, Japan) for 20 min and then frozen. Sections perpendicular to the corneal surface were cut at eight microns on a Minitome cryostat (International Equipment Corporation, Needham, MA) and collected onto chrome-alum-gelatin coated slides.

Sections were washed three times in 0.1 M PBS at RT, permeabilized for 20 min in ice-cold methanol, and then washed three times in 0.1 M PBS – 0.3% Triton X-100 (TX). Sections were incubated for 20 min at RT in 10% normal horse serum (Vector Laboratories, Burlingame, CA*)* in 0.1 M PBS – 0.3% TX. Alternate slides were then incubated overnight at 4 °C in either panPMCA antibody (5F10; 1:5,000 dilution) or in PMCA4 isoform-specific antibody (JA9; 1:1,000 dilution). The vehicle in both cases was solution supplemented with 10% normal horse serum and 0.3% TX. Monoclonal antibodies 5F10 and JA9 were a generous donation (Brent Rolland, Executive Vice President; Affinity BioReagents, Inc., Golden, CO) and have been previously characterized [49,55,56]. Sections were then washed three times in 0.1 M PBS – 0.3% TX and incubated with Fluorescein (FITC)–conjugated, horse-anti-mouse IgG (1:100; Vector Laboratories) for 60 min at RT in a humidified chamber. After a final wash, the slides were coverslipped with SlowFade Mounting Medium (Molecular Probes*)*. Negative control sections of CE were incubated with non-immunized mouse IgG in place of the primary antibody.

### Microscopy

Tissue sections were examined using a Leitz Laborlux - S (Wetzlar, Germany) microscope fitted with an FITC cube. Images were captured by epifluorescence using a Magnifier CCD camera (Olympus America, Inc., Center Valley, PA) and Magnafire SP V1.0x5 (Optronics®, Goleta, CA) and ImagePro® Express V4.0 (Media Cybernetics, Bethesda, MD) software, and processed using Adobe Photoshop (Adobe Systems Incorporated, Orem, UT).

**Figure 1:**
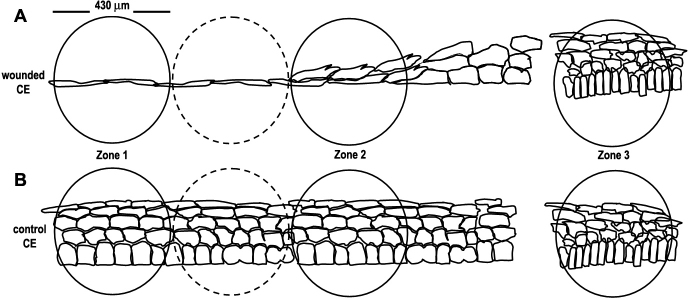
Quantitation of plasma membrane calcium-ATPase isoform 4 (PMCA4) expression in rabbit corneal epithelium. PMCA4 localization in basal epithelial cells of wounded CE **(A)** and control CE **(B)** was analyzed quantitatively with reference to three defined zones.

**Figure 2:**
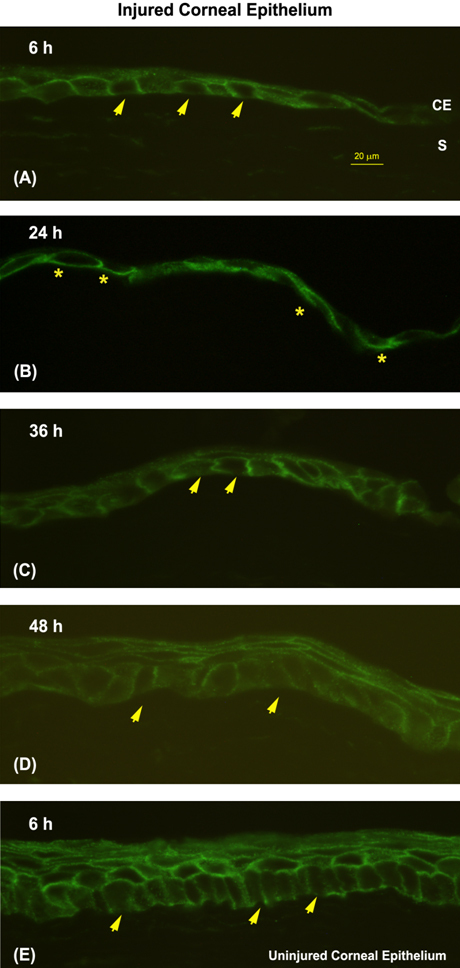
Redistribution of plasma membrane calcium-ATPase isoform 4 (PMCA4) in basal epithelial cells at the wound margin during corneal reepithelialization. The pattern of PMCA4 IR is shown in the wound margin at 6 h (**A**), 24 h (**B**), 36 h (**C**), and 48 h (**D**), and in the control cornea at 6 h (**E**). The presence (asterisks) or absence (arrows) of basal plasma membrane staining in representative cells is shown (corneal epithelium [CE]; stroma [S]). Bars in **A** represent 20 µm and apply to all panels.

**Figure 3:**
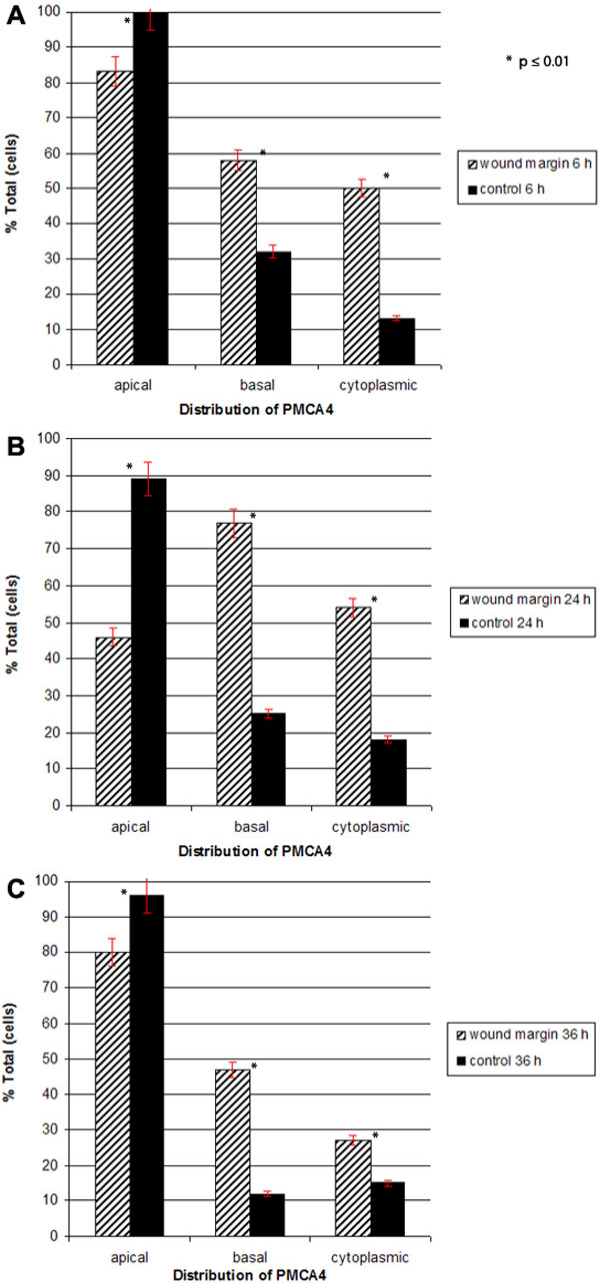
Quantitative analysis of plasma membrane calcium-ATPase isoform 4 (PMCA4) distribution in basal epithelial cells at the wound margin during reepithelialization. The percentage of basal epithelial cells that exhibited PMCA4 staining in the apical plasma membrane, basal plasma membrane and cytoplasm in wounded and control corneas at 6 h (**A**), 24 h (**B**), and 36 h (**C**) is shown. Note in particular, the prominent redistribution of PMCA4 staining from apical to basal cell membranes in the wounded eye between 6 and 24 h as well as the increase in cytoplasmic staining in wounded versus controls eyes at 6 and 24 h. The asterisk (*) represents a p value less than or equal to 0.01.

Early in this investigation, it was noted that the pattern of PMCA4 expression in the basal epithelial cell plasma membrane of wounded CE was altered compared to that observed in control basal cells. Therefore, we sought to quantify these changes in experimental corneas at each time point examined during the wound healing process. Three zones were defined for this purpose (Figure 1). Zone 1 (wound margin) in the experimental eye was defined as one microscopic field of view (430 μm in diameter at a magnification of 400×) beginning at the wound edge and continuing peripherally across the CE. Zone 2 (juxtamarginal zone) comprised the microscopic field of view lying two fields of view peripheral to Zone 1. Zone 3 contained the limbus. Equivalent zones were defined for similar regions of the contralateral (control) eye. The only Zone where marked PMCA4 changes were observed was Zone 1, and these data are presented here (Figure 2 and Figure 3). For the purposes of this study, a basal cell in the wounded area was considered to be any cell of any shape that was resting on the basal lamina. The number of basal epithelial cells that stained positively for PMCA4 immunoreactivity [46] in the apical plasma membrane, basal plasma membrane, or cytoplasm were counted for tissue sections from each wounded cornea and at each time interval. For each animal, analysis was performed on a minimum of three tissue sections from the wounded and paired control eye. As noted above, the experiments were repeated using 3 animals at the 6 h, 24 h, and 36 h post-wounding time points. Two animals were examined at 48 h post-wounding. Bar graphs in Figure 3 present the combined data from three different animals at the 6 h, 24 h, and 36 h post-wounding time points. The number of basal epithelial cells that expressed each phenotype was calculated as the percentage of the total number of basal cells in that zone.

## Results

### Plasma membrane calcium-ATPase (PMCA) expression and distribution in normal rabbit corneal epithelium

**Figure 4:**
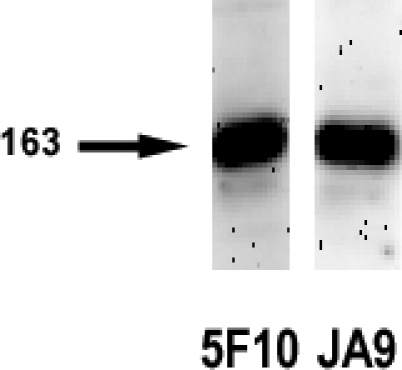
Immunoblot analysis of plasma membrane calcium-ATPase (PMCA) in rabbit corneal epithelium. Blot lanes were loaded with equal aliquots of whole cell lysate. The panPMCA antibody was used at 0.65 mg/ml, and the PMCA4 antibody was used at 0.4 mg/ml (data presented here is from a single animal and is representative of immunoblot studies of the CE from three different normal animals).

Western blotting and PCR analysis were used to characterize PMCA4 expression in rbCE. In immunoblotting studies (Figure 4), both the panPMCA and PMCA4 isoform-specific antibody labeled an intense band at approximately 163 kDa. In rat PMCA4 at site A, a 36 bp exon can undergo alternative splicing [38]. Direct sequencing of the site A product from rbCE revealed that the 36 bp exon was inserted. Rabbit CE thus expresses splice variant PMCA4x (designation is based on the currently accepted nomenclature as described by Kamagate, et al. [38]). This sequence has 100% identity with PMCA4x from human red blood cell (NCBI NM 001684.1) and retinal pigment epithelium [51].

**Figure 5:**
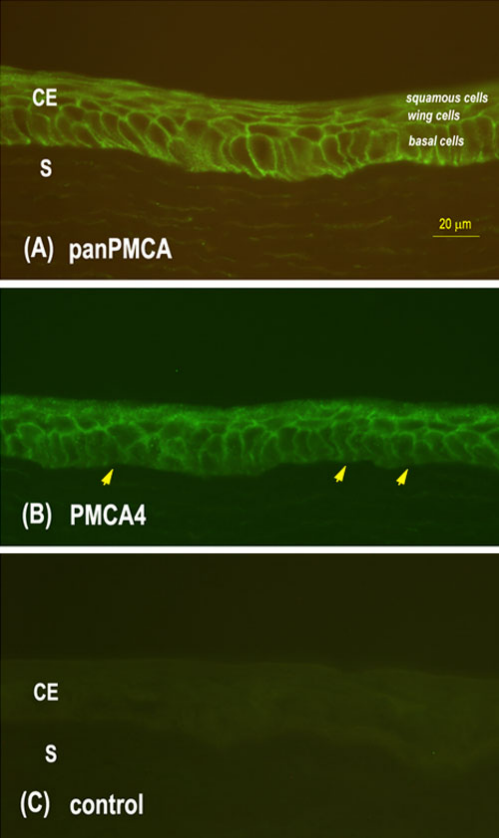
Localization of plasma membrane calcium-ATPase (PMCA) in rabbit corneal epithelium (CE). **A**: Immunostaining with pan-PMCA antibody revealed strong PMCA labeling in all layers of the CE. The staining was associated primarily with the plasma membranes with diffuse cytoplasmic staining in some cells. The corneal stroma (S) was unstained. **B**: The pattern and intensity of immunoreactivity seen with PMCA4 isoform-specific antibody was similar to that seen with the panPMCA antibody, except that PMCA4 labeling is absent (arrows) from most basal cell plasma membranes adjacent to the stroma. **C**: Control section incubated with nonimmune mouse IgG revealed an absence of staining.

Immunohistochemical staining of normal rbCE with both the panPMCA Ab and the PMCA4 isoform-specific Ab revealed strong labeling of plasma membranes in all cell layers and all regions of the CE (Figure 5). The corneal stroma was unstained. The distributions of panPMCA IR and PMCA4 IR were similar with the notable exception that in a large percentage of basal epithelial cells PMCA4 staining was absent from these cells’ basal (i.e., stromal facing) plasma membranes (Figure 5B). Immunohistochemical control sections incubated with nonimmunized mouse IgG in place of primary Ab were unstained (Figure 5C).

### Distributional changes in plasma membrane calcium-ATPase isoform four (PMCA4) expression during corneal reepithelialization of injured rabbit corneal epithelium

Analysis of PMCA4 IR demonstrated substantial temporal and spatial changes in PMCA4 distribution in basal epithelial cells at the wound margin during corneal epithelial wound healing. In contrast, the patterns of PMCA4 distribution basal cells of the juxtamarginal zone and corneoscleral limbus were unchanged from control corneas.

At 6 h post epithelial wounding, the wound margin consisted of 1 or 2 layers of cuboidal or squamous cells (Figure 2A). Approximately 83% of the basal cells located at the wound margin demonstrated apical staining, compared to 100% of basal epithelial cells from the control eye in the same animal treated with PBS (Figure 3A). Approximately 58% of the basal cells in injured CE showed PMCA4 IR along the basal membrane, compared to 32% of the basal cells in control CE. Fifty percent of the marginal zone basal cells in wounded CE contained cytoplasmic staining, as compared to only 13% of basal cells in nonwounded CE.

At 24 h post wounding, the cells at the wound margin comprised a monolayer of long, flattened cells (Figure 2B). By 24 h following injury the percentages of basal cells that showed apical or basal plasma membrane labeling were essentially reversed (Figure 3B). Approximately 46% of cells at the wound margin demonstrated apical staining compared to 89% of basal cells in the corresponding region of the control cornea. Seventy-seven percent of cells in the area of the wound margin exhibited IR along the basal cell membrane, as compared to 25% in control CE. The percentage of basal cells in either eye that demonstrated cytoplasmic PMCA4 immunoreactivity was relatively unchanged from 6 h.

At 36 h post injury, the wound was closed and the previously denuded area was completely covered by a partially stratified epithelium consisting of 2–3 cell layers (Figure 2C). The polarity of PMCA4 labeling in basal epithelial cells had reversed from that seen at 24 h (Figure 2B,C) and began to more closely resemble that seen in normal and control CE (Figure 2E). Eighty percent of the basal cells in the area of the former wound margin showed apical staining as compared to 96% of the cells in the comparable region of the control cornea (Figure 3C). Forty-seven percent of basal cells in the formally denuded area and 12% of the basal cells in the control CE demonstrated basal staining. Finally, approximately 27% of cells in the area of the former wound area and 12% of those cells in the corresponding area of the control cornea showed cytoplasmic PMCA4 labeling.

At 48 h post injury, the process of re-stratification had continued to a point where the CE consisted of a well defined layer of columnar-shaped basal cells, a layer of wing cells, and 1–2 layers of flattened squamous cells (Figure 2D). The plasma membranes of all the cells in all layers were positive for panPMCA labeling (data not shown). The pattern of PMCA4 staining in basal cells was indistinguishable from that seen in basal cells in control CE and normal CE.

### Effect of siRNA_PMCA4_ transfection on wound healing using the hTCEpi cell line

**Figure 6:**
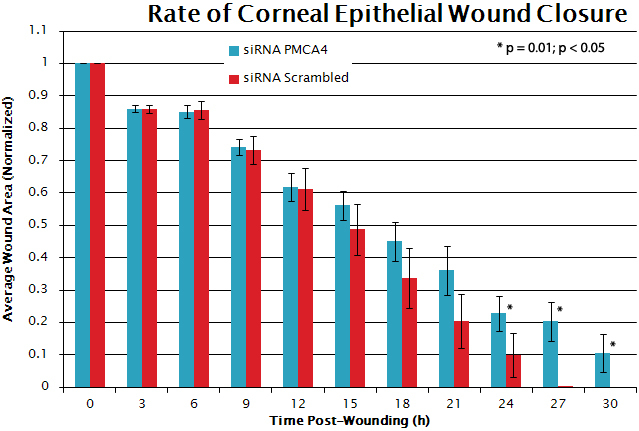
Graphical representation of rate of corneal epithelial cell culture wound closure in siRNA transfected cultures. Wounded siRNA_PMCA4_ (blue) transfected hTCEpi cells fail to close the wound defect in monolayer of hTCEpi cell cultures. hTCEpi cells transfected with siRNA_Scrambled_ (red) do migrate over the wounded area and close the wound defect earlier than hTCEpi cells where PMCA4 expression has been reduced. The asterisk (*) represents a p value equal to 0.01.

**Figure 7:**
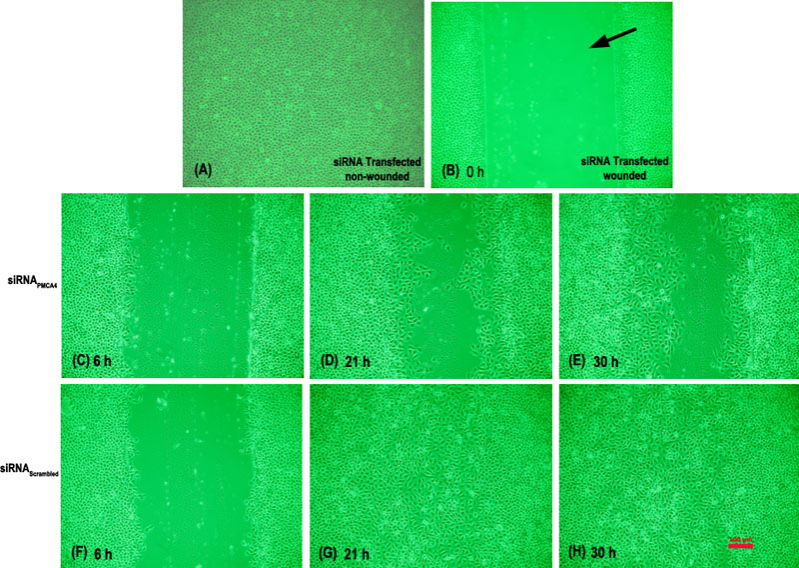
Photomicrograph of representative siRNA transfected hTCEpi cultures. This figure shows photomicrographs of representative siRNA transfected hTCEpi cell cultures: (**A**) non-wounded; (**B**) wounded at 0 h post-wounding; (**C**), (**D**), and (**E**) are siRNA_PMCA4_ transfected cell cultures at 6 h, 21 h, and 30 h post-wounding, respectively; (**F**), (**G**), and (**H**) are siRNA_Scrambled_ transfected cell cultures for the corresponding respective time intervals. At 21 and 30 h post-wounding are siRNA_PMCA4_ CE cells fail to close the denuded (i.e., wounded) area as compared to siRNA_Scrambled_ transfected cells where the wound is closed at 21 h. Bar in **H** represents 100 µm and applies to all panels.

An siRNA construct against exon 17 of PMCA4 and a scrambled construct with no known homology to any human mRNA were used to test the hypothesis that knockdown of PMCA4 expression would adversely affect CE wound healing. Overall, the rate of wound closure was slower in siRNA_PMCA4_ transfected cultures as compared to siRNA_Scrambled_ transfected (control) cultures (Figure 6). Some control cultures were completely healed as early as 21 h post-injury, and all control cultures were healed between 24 and 27 h (Figure 6 and Figure 7). In contrast, in all siRNA_PMCA4_ transfected cultures CE cells failed to completely close the wound defect at the maximum measured time point of 30 h (Figure 6 and Figure 7E).

## Discussion

### Plasma membrane calcium ATPase expression in the normal rabbit CE

The results of this study have shown for the first time, using three different methods (PCR, immunoblotting, and immunohistochemistry) the presence of PMCA in the normal rabbit corneal epithelium. The data mirror results from our previous report of PMCA expression and distribution in human CE [47], as well as data from other investigators reporting the presence of PMCA in rat [57], bovine [58], and mouse CE [41]. Sequencing of the PCR DNA from rabbit revealed that PMCA4x is the splice variant in rabbit CE at site A. PMCA4x is the same splice variant expressed in human corneal epithelium [47,48]. Immunohistochemical staining with a panPMCA antibody demonstrates that PMCAs are expressed by all cells in the normal rabbit CE and limbus, and that PMCAs are located mainly in the plasma membrane; however, the presence in some cells of diffuse cytoplasmic staining suggests that some PMCAs are associated with cytoplasmic membranes. It is suggested that that PMCAs, via their ability to extrude intracellular calcium into the extracellular space, play key roles in critical calcium-dependent cell behaviors in this continuously renewing epithelium including, proliferation, migration, adhesion, and differentiation [20-25].

### Redistribution of PMCA4 and possible functional roles for PMCA4 during corneal epithelial wound healing

The data presented in this investigation show that PMCA4 IR exhibited a marked change in distribution of epithelial cells at the wound margin during CE wound healing. In normal and control rabbit corneal epithelium, PMCA4 IR was expressed in basal epithelial cells in a strongly “polarized” fashion with the isoform IR most prominent along the plasma membranes at the apices and lateral plasma membranes of the basal cells and weakly (or not at all in most cells) along the plasma membranes facing the basal lamina. This polarity of PMCA4 immunostaining “reverses” during epithelial wound healing. That is, the basal cells near the wound margin express more PMCA4 along the basal membrane than apically, with a concurrent increase in cytoplasmic staining. This pattern of IR then reverts back to control (or non-injured) conditions soon after the denuded area is closed. A similar pattern of protein redistribution in mouse CE was found for fodrin by Takahashi et al. [59]. Fodrin is an actin-binding protein, and forms a complex with E-cadherin, a Ca^2+-^dependent cell-cell adhesion protein [60]. It is well known that the cytoskeleton protein actin plays a major role in cell migration. This redistribution of fodrin and interaction with E-cadherin sets the stage to evaluate the possible functional role(s)/interaction(s) of PMCA4 in cell migration and wound healing.

During the latent phase of wound healing, basal cells at the wound margin undergo extensive cellular and subcellular reorganization including loss of columnar appearance, increase in cell volume and breakdown of hemisdesmosomal attachments to the stroma [10-13]. During the migration phase of CE wound healing, cells at the leading edge of the wound margin begin to migrate across the denuded area. These cells pull more peripheral cells behind them as a continuous epithelial sheet until the wound defect is covered [14-17]. We hypothesize that the redistribution of PMCA4 observed during the migration phase may relate to altered Ca^2+^ needs required for cell movement and changes in the stability of cell-to-cell or cell-to-matrix junctions. For example, E-cadherin is found in cell membranes of corneal epithelial cells [61]. In the classical cadherin model, extracellular Ca^2+^ interacts within pockets located in E-cadherins to form rigid E-cadherin dimers. Under the influence of additional Ca^2+^, these dimers bind adjacent cells together by forming E-cadherin oligomers [25]. PMCA4 may function to stabilize these adhesions by pumping Ca^2+^ into the junctional spaces. In the present study, PMCA4 redistributes from the plasma membrane to the cytoplasm (Figure 2 and Figure 3); the resulting decrease in extracellular Ca^2+^ available to interact with E-cadherin may result in the more flexible cell-to-cell junctional complexes needed for epithelial sheet migration. A prior study by Amino et al. [41] yielded results compatible with this hypothesis. These investigators demonstrated PMCAs redistribute from cell membrane caveolae to cytoplasmic membranes during re-epithelialization of mouse CE following wounding.

The finding that basal cells at the wound margin express more PMCA4 in the basal plasma membrane than “control” basal cells may suggest a role for PMCA in cell-to-extracellular matrix adhesion. Ca^2+^ in the ECM at the site of focal adhesions interacts with calpain, which then cleaves integrins at the trailing ends of migrating cells [20]. In contrast, intracellular Ca^2+^ stabilizes the cytoplasmic components of integrins near the advancing edges of migrating cells [20]. Thus, PMCA4 shifted to the basal plasma membrane of migrating basal epithelial cells may help direct these opposing processes. This idea gains support from one study in which Brundage, et al. [62] demonstrated regional changes in Ca^2+^ concentration both at the leading and retracting edges of migrating eosinophils. In the present study, when PMCA4 expression was knocked down via siRNA_PMCA4_ transfection, migration slowed and cells failed to close the wound defect within a comparable time interval to control cultures. These results when correlated to the redistribuiton of PMCA4 to the portion of the basal cell plasma membrane adjacent to the corneal stroma during cell migration suggest of a role for PMCA4 in the regulation of Ca^2+^-handling in the formation of cell-to-extracellular matrix adhesion during migration. PMCA4 may also be involved with regulation of Ca^2+^-handling needed for fodrin-actin interaction during cell migration as indicated by the pattern of fodrin redistribution during CE being similar to that of PMCA4 in the present study [59].

The basal cells of CE are the only cells in CE that are capable of a mitogenic or proliferative response. CE proliferation (and regeneration) is affected by a host of cytokines, such as epidermal growth factor (EGF) [4]. Following injury to CE, EGF synthesis increases and EGF stimulates wound closure via a variety of signaling pathways, including cascades that activate calcium transients [63]. More recently, canonical transient receptor potential protein isoform TRPC4 knockdown has been shown to suppress EGF - induced store-operated Ca^2+^ channel activation and growth in human CE [64]. This suggests that TRPC4 expression is required for EGF to be maximally effective through inositol 1,4,5 triphosphate (IP_3_) –linked pathways. Interestingly, Sgambato-Fuare et al. [65], showed the interaction of the Homer scaffold protein, Homer-1/Ania-3, with PMCAs suggesting coupling of PMCAs to IP_3_ receptor Ca^2+^ channels. Taken together with the results presented herein, it is reasonable to hypothesize a connection between TRPC4 and PMCA via IP_3_ –linked pathways that may affect mitogenic response in CE.

At the present time the nature of the mechanism that results in a shift of PMCA4 from apical to basal plasma membranes in incompletely understood. However, it is reasonable to suggest that alternative splicing of PMCA4 primary transcripts may be involved. Support for this idea comes from several experimental studies showing that PMCAs may be “selectively” targeted to different areas of the cell plasma membrane. Chicka et al. [66] have shown that alternative splicing of PMCA2 at splice site A (near the NH_2_-terminus along the first intracellular loop) alters its membrane targeting in MDCK (Madin-Darby Canine kidney epithelial) epithelial cells. These data demonstrated that alternative splicing at the A site involved a PDZ-protein binding domain such that PMCA2w, containing the full length spliced-in insert was targeted to apical and basal cell membranes, but the PMCA2x and z variants, containing a partial insert or no insert, respectively, were selectively targeted to the basal lateral membrane. Additional evidence suggests that PMCA b-splice variants (at the C splice site) interact with PMCA-interacting single PDZ protein for the sorting of PMCAs to or from the plasma membrane [67,68]. This is interesting in that in prior experiments in our laboratory, human CE has been shown to express PMCA4x,b [47,48]; here in this investigation rabbit CE expresses PMCA4x (the C splice site variant in rabbit is yet undetermined). However, it is not clear at the present time if a change in alternative splice variant expression occurs during corneal epithelial wound healing.

This study shows that PMCA4 is strongly expressed in rabbit corneal epithelium and that the pattern of PMCA4 IR markedly changes during the process of corneal epithelial wound healing. The functional significance of the change in PMCA4 localization is not resolved by this study, however, the results here suggest a role for PMCA4 in migration during CE wound healing. Most certainly Ca^2+^-handling requirements of corneal epithelial cells are altered during reepithelialization, a process that comprises morphological changes, migration, proliferation and differentiation. PMCA4 redistribution would allow the cells of the CE to maintain different “regional” baseline levels of [Ca^2+^]_i_ and to selectively respond to signals regulating [Ca^2+^]_i_ and junctional levels of Ca^2+^ during CE wound healing. To fully understand calcium handling by the corneal epithelium during the wound healing process, the transporters catalyzing calcium efflux must be characterized. The present work has begun this process by examining PMCA4 expression and distribution in rabbit corneal epithelial wound healing, and the knockdown of PMCA4 expression in corneal epithelial wound healing in vitro. The stage is now set for additional studies to further characterize the functional role(s) of each PMCA isoform in wound healing of stratified epithelium. These functional experiments might include the selective knockdown of additional PMCA isoform expression in corneal epithelial cells during adhesion and migration studies, and may also investigate possible interactions of PMCAs with fodrin, actin and E-cadherins.
